# The interactions of ZDHHC5/GOLGA7 with SARS-CoV-2 spike (S) protein and their effects on S protein’s subcellular localization, palmitoylation and pseudovirus entry

**DOI:** 10.1186/s12985-021-01722-w

**Published:** 2021-12-27

**Authors:** Xiao-Tao Zeng, Xiao-Xi Yu, Wei Cheng

**Affiliations:** Division of Respiratory and Critical Care Medicine, Respiratory Infection and Intervention Laboratory of Frontiers Science Center for Disease-Related Molecular Network, State Key Laboratory of Biotherapy, West China Hospital of Sichuan University, Chengdu, 610041 China

**Keywords:** SARS-CoV-2, Spike protein, ZDHHC5/GOLGA7, APT2, Virus-host interaction

## Abstract

**Background:**

Severe acute respiratory syndrome coronavirus 2 (SARS-CoV-2) spike (S) protein determines virus entry and the palmitoylation of S protein affects virus infection. An acyltransferase complex ZDHHC5/GOGAL7 that interacts with S protein was detected by affinity purification mass spectrometry (AP-MS). However, the palmitoylated cysteine residues of S protein, the effects of ZDHHC5 or GOLGA7 knockout on S protein’s subcellular localization, palmitoylation, pseudovirus entry and the enzyme for depalmitoylation of S protein are not clear.

**Methods:**

The palmitoylated cysteine residues of S protein were identified by acyl-biotin exchange (ABE) assays. The interactions between S protein and host proteins were analyzed by co-immunoprecipitation (co-IP) assays. Subcellular localizations of S protein and host proteins were analyzed by fluorescence microscopy. *ZDHHC5* or *GOGAL7* gene was edited by CRISPR-Cas9. The entry efficiencies of SARS-CoV-2 pseudovirus into A549 and Hela cells were analyzed by measuring the activity of *Renilla* luciferase.

**Results:**

In this investigation, all ten cysteine residues in the endodomain of S protein were palmitoylated. The interaction of S protein with ZDHHC5 or GOLGA7 was confirmed. The interaction and colocalization of S protein with ZDHHC5 or GOLGA7 were independent of the ten cysteine residues in the endodomain of S protein. The interaction between S protein and ZDHHC5 was independent of the enzymatic activity and the PDZ-binding domain of ZDHHC5. Three cell lines HEK293T, A549 and Hela lacking ZDHHC5 or GOLGA7 were constructed. Furthermore, S proteins still interacted with one host protein in HEK293T cells lacking the other. ZDHHC5 or GOLGA7 knockout had no significant effect on S protein’s subcellular localization or palmitoylation, but significantly decreased the entry efficiencies of SARS-CoV-2 pseudovirus into A549 and Hela cells, while varying degrees of entry efficiencies may be linked to the cell types. Additionally, the S protein interacted with the depalmitoylase APT2.

**Conclusions:**

ZDHHC5 and GOLGA7 played important roles in SARS-CoV-2 pseudovirus entry, but the reason why the two host proteins affected pseudovirus entry remains to be further explored. This study extends the knowledge about the interactions between SARS-CoV-2 S protein and host proteins and probably provides a reference for the corresponding antiviral methods.

## Introduction

SARS-CoV-2 is a highly transmissible and pathogenic beta-coronavirus and causes the coronavirus disease 19 (COVID-19) pandemic, threatening human health and public safety. SARS-CoV-2 contains a single-stranded positive-sense RNA genome that encodes at least 29 proteins, including four typical structural proteins, spike (S), envelope (E), membrane (M) and nucleocapsid (N) proteins [1–3]. However, the pathogenic mechanism of SARS-CoV-2 including the interaction between virus and host remains to be further explored.

SARS-CoV-2 S protein is the major target of virus-neutralizing antibodies, forming a trimer on the surface of the virus particle and binding to the receptor to initiate the viral replication cycle [4]. Each S protomer comprises the S1 and S2 subunits and activation of S protein requires cleavage of S1/S2 by furin-like protease and undergoes a conformational change from prefusion to postfusion [5]. S protein undergoes palmitoylation and alteration of the ten cysteine residues in the endodomain (cytoplasmic tail) of S protein decreased the efficiency of syncytium formation, cell–cell fusion and pseudotyped SARS-CoV-2 infectivity [6]. The palmitoylations of S proteins from other coronaviruses have also been reported. For example, palmitoylation of the cysteine-rich endodomain of S protein from SARS-CoV (severe acute respiratory syndrome coronavirus) is important for spike-mediated cell fusion [7]. The palmitoylation of S protein from murine coronavirus is essential for virion assembly and infectivity [8].

Protein palmitoylation is a dynamic and reversible post-translational modification that is catalyzed by 23 mammalian palmitoyltransferases ZDHHCs and is reversed by several acyl protein thioesterases (such as APT1 and APT2) [9, 10]. Palmitoylation dynamically regulates different aspects in the life of a protein, including stability, localization, interactome and function, playing critical roles in cellular physiology. Each of ZDHHCs intracellular domains contains a conserved Asp-His-His-Cys (DHHC) motif as the catalytic center [11]. Other accessory proteins such as GOLGA7 (or GCP16) are essential components of cell palmitoylation system and can regulate specific ZDHHC enzyme activity, stability and transport [12]. Virus protein palmitoylation is usually very important for viral protein function and viral replication. Therefore, ZDHHCs enzyme necessary for palmitoylation of viral proteins can be used as a potential drug target [13].

Two high confidence human proteins ZDHHC5 and GOLGA7 that interacted with SARS-CoV-2 S protein were identified by AP-MS [14]. ZDHHC5 and accessory protein GOLGA7 form a mutually stable acyltransferase complex [15]. ZDHHC5 can palmitoylate many protein substrates and participate in some important physiological processes, such as fatty acid uptake and immune response [16, 17]. ZDHHC5 usually interacts with substrate proteins through its own PDZ (PSD-95/Discs-large/ZO-1 homology) binding domain and substitution of the cysteine residue in DHHC motif by a serine residue leads to enzyme inactivation [18]. However, the palmitoylated cysteine residues of S protein, the effects of ZDHHC5 or GOLGA7 knockout on S protein’s subcellular localization, palmitoylation, pseudovirus entry, and the enzyme for depalmitoylation of S protein are not clear. In this study, we mainly identified the palmitoylated cysteine residues of S protein, investigated the effects of ZDHHC5 or GOLGA7 knockout on S protein’s subcellular localization, palmitoylation, pseudovirus entry and analyzed the interactions of S protein with two depalmitoylases APT1/APT2.

## Methods

### Cell lines

Human embryonic kidney (HEK) 293 T cells provided by Prof. Hai-Yan Ren, human lung cancer A549 cells purchased from National Collection of Authenticated Cell Cultures and human cervical carcinoma Hela cells provided by Prof. Feng Shao were grown at 37 °C in 5% CO2 in Dulbecco’s modified Eagle’s medium (DMEM, Gibco) supplemented with 10% fetal bovine serum (FBS). HEK293T cells were used for the ABE and co-IP assays, since the transfection efficiency of HEK293T cells is very high. A549 and Hela cells were used for the fluorescence microscopy and SARS-CoV-2 spike-mediated pseudovirus entry assays, since the two kinds of cells adhered well to the surface of coverslips placed in 6-well plates and on the bottom of 96-well white plates.

### Plasmid construction

Total RNA was extracted from HEK293T cells with Trizol reagent (Invitrogen) and was then used for the synthesis of single-stranded cDNA by reverse transcription. The plasmid pcDNA3.1-*S* expressing SARS-CoV-2 isolate Wuhan-Hu-1 (GenBank accession number NC_045512.2) S protein was provided by Prof. Ai-Ping Tong. Different kinds of nucleic-acid fragments were amplified with the corresponding primers (Table 1). The plasmids used for ABE, co-IP and fluorescence microscopy were constructed. All constructs used in this study were confirmed by DNA sequencing. The operations are as follows.

**Table 1:** Primers used in this study

Name	Sequence(5'to 3')^a^	Usage
*S*-F2	CTAGCGTTTAAACTTAAGCTTATGTTTGTTTTTCTTGTTTTATTGCCACTAGTCTCTAGTCAGTGCGTGAACCTGACCACA *(HindIII)*	pcDNA3.1-*S-HA*, pcDNA3.1-*SC10A-HA*/Co-IP, ABE
*S-HA*-R	TGCTGGATATCTGCAGAATTCTTAAGCGTAATCTGGAACATCGTATGGGTACATGGTGTAGTGCAGCTTCACGCC *(EcoRI)*	
*SA1235C-*F	TATCGTGATGGTGACCATCATGCTGTGCGCTATGACATCC	pcDNA3.1-*SA1235C-HA/*ABE
*SA1235C-*R	GGATGTCATAGCGCACAGCATGATGGTCACCATCACGATA	
*SA1236C-*F	TATCGTGATGGTGACCATCATGCTGGCCTGTATGACATCC	pcDNA3.1-*SA1236C-HA/*ABE
*SA1236C-*R	GGATGTCATACAGGCCAGCATGATGGTCACCATCACGATA	
*SA1240C-*F	CATCATGCTGGCCGCTATGACATCCTGCGCTTCTGCCCTG	pcDNA3.1-*SA1240C-HA/*ABE
*SA1240C-*R	CAGGGCAGAAGCGCAGGATGTCATAGCGGCCAGCATGATG	
*SA1241C-*F	CATCATGCTGGCCGCTATGACATCCGCCTGTTCTGCCCTG	pcDNA3.1-*SA1241C-HA/*ABE
*SA1241C-*R	CAGGGCAGAACAGGCGGATGTCATAGCGGCCAGCATGATG	
*SA1243C-*F	GCTGGCCGCTATGACATCCGCCGCTTCTTGCCTGAAGGGC	pcDNA3.1-*SA1243C-HA/*ABE
*SA1243C-*R	GCCCTTCAGGCAAGAAGCGGCGGATGTCATAGCGGCCAGC	
*SA1247C-*F	GACATCCGCCGCTTCTGCCCTGAAGGGCTGCGCTAGCGCT	pcDNA3.1-*SA1247C-HA/*ABE
*SA1247C-*R	AGCGCTAGCGCAGCCCTTCAGGGCAGAAGCGGCGGATGTC	
*SA1248C-*F	GACATCCGCCGCTTCTGCCCTGAAGGGCGCCTGTAGCGCT	pcDNA3.1-*SA1248C-HA/*ABE
*SA1248C-*R	AGCGCTACAGGCGCCCTTCAGGGCAGAAGCGGCGGATGTC	
*SA1250C-*F	TCTGCCCTGAAGGGCGCCGCTAGCTGTGGCTCCGCCGCTA	pcDNA3.1-*SA1250C-HA/*ABE
*SA1250C-*R	TAGCGGCGGAGCCACAGCTAGCGGCGCCCTTCAGGGCAGA	
*SA1253C-*F	TCTGCCCTGAAGGGCGCCGCTAGCGCTGGCTCCTGCGCTA	pcDNA3.1-*SA1253C-HA/*ABE
*SA1253C-*R	TAGCGCAGGAGCCAGCGCTAGCGGCGCCCTTCAGGGCAGA	
*SA1254C-*F	CTGAAGGGCGCCGCTAGCGCTGGCTCCGCCTGTAAGTTTG	pcDNA3.1-*SA1254C-HA/*ABE
*SA1254C-*R	CAAACTTACAGGCGGAGCCAGCGCTAGCGGCGCCCTTCAG	
*ZDHHC5*-F	CTTGGTACCGAGCTCGGATCCATGCCCGCAGAGTCTGGAAAG *(BamHI)*	pcDNA3.1-*ZDHHC5-3Flag*/co-IP
*ZDHHC5-3Flag*-R	TGCTGGATATCTGCAGAATTCTCACTTATCGTCGTCATCCTTGTAATCGATCTTATCGTCGTCATCCTTGTAATCTCCCTTATCGTCGTCATCCTTGTAATCCACCGAAATCTCATAGGTGG *(EcoRI)*	
*GOLGA7*-F	CTTGGTACCGAGCTCGGATCCATGAGGCCGCAGCAGGCGCC *(BamHI)*	pcDNA3.1-*GOLGA7-3Flag*/co-IP
*GOLGA7-3Flag*-R	TGCTGGATATCTGCAGAATTCTTACTTATCGTCGTCATCCTTGTAATCGATCTTATCGTCGTCATCCTTGTAATCTCCCTTATCGTCGTCATCCTTGTAATCTCTTCCACTGCTCATGCCTC *(EcoRI)*	
*ZDHHC5-C134S-*F	TGTGGAGGAATTTGATCATCACTCCCCCTGGGTGAATAAC	pcDNA3.1-*ZDHHC5-C134S-3Flag*/co-IP
*ZDHHC5-C134S-*R	GTTATTCACCCAGGGGGAGTGATGATCAAATTCCTCCACA	
*ZDHHC5△PDZ-3Flag-*R	TGCTGGATATCTGCAGAATTCTCACTTATCGTCGTCATCCTTGTAATCGATCTTATCGTCGTCATCCTTGTAATCTCCCTTATCGTCGTCATCCTTGTAATCGGTGGTACCACCAACCCCTG *(EcoRI)*	pcDNA3.1-*ZDHHC5△PDZ-3Flag*/co-IP
*ZDHHC5△PDZ-*R	TTATCTAGATCCGGTGGATCCTCAGGTGGTACCACCAACCCCTG *(BamHI)*	pDsRed2-*ZDHHC5△PDZ*/localization
*S*-F	TCAGATCTCGAGCTCAAGCTTATGTTTGTTTTTCTTGTTTTATTGCCACTAGTCTCTAGTCAGTGCGTGAACCTGACCACA *(HindIII)*	pEGFP-*S*/localization
*S*-R	CACCATGGTGGCGATGGATCCGGTGTAGTGCAGCTTCACGCC *(BamHI)*	
*SC10A*-R	CACCATGGTGGCGATGGATCCGGTGTAGTGCAGCTTCACGCCCTTCAGCACTGGCTCGGAATCGTCCTCATCAAACTTAGCGGCGGAGCCAGCGCTAGCGGCGCCCTTCAGGGCAGAAGCGGCGGATGTCATAGCGGCCAG *(BamHI)*	pEGFP-*SC10A*/localization
*ZDHHC5*-F2	TCGAGCTCAAGCTTCGAATTCTATGCCCGCAGAGTCTGGAAAG *(EcoRI)*	pDsRed2-*ZDHHC5*/localization
*ZDHHC5*-R2	TTATCTAGATCCGGTGGATCCTCACACCGAAATCTCATAGG *(BamHI)*	
*GOLGA7*-F2	TCGAGCTCAAGCTTCGAATTCTATGAGGCCGCAGCAGGCGCC *(EcoRI)*	pDsRed2-*GOLGA7*/colocalization
*GOLGA7*-R2	TTATCTAGATCCGGTGGATCCTTATCTTCCACTGCTCATGC *(BamHI)*	
*ZDHHC5-sgRNA-*F	CACCGGAACCACGTGAAATCCCGTG	lentiCRISPRv2-*ZDHHC5 sgRNA*/gene editing
*ZDHHC5-sgRNA-*R	AAACCACGGGATTTCACGTGGTTCC	
*ZDHHC5-*F3	TGTATTGGTCGCCGGAACTA	PCR
*ZDHHC5-*R3	CAAGATCACGCCACTGGATG	
*ZDHHC5-seq-*F	CACGTATCCTTCAAGGCC	Sequencing
*GOLGA7-sgRNA-*F	CACCGGGCGGCCAGTCATATCTCGA	lentiCRISPRv2-*GOLGA7 sgRNA*/gene editing
*GOLGA7-sgRNA-*R	AAACTCGAGATATGACTGGCCGCCC	
*GOLGA7-*F3	CATTGCCTGTTCTGCTTGCA	PCR
*GOLGA7-*R3	CCCTCATGCCCAAAGATGGT	
*GOLGA7-seq-*F	GTTTTGGTAACTTAGGCCCAG	Sequencing
*APT1-*F	CTTGGTACCGAGCTCGGATCCATGTGCGGCAATAACATGTC *(BamHI)*	pcDNA3.1-*APT1-3Flag*/co-IP
*APT1-3Flag-*R	TGCTGGATATCTGCAGAATTCTCACTTATCGTCGTCATCCTTGTAATCGATCTTATCGTCGTCATCCTTGTAATCTCCCTTATCGTCGTCATCCTTGTAATCATCAATTGGAGGTAGGAGTT *(EcoRI)*	
*APT2-*F	CTTGGTACCGAGCTCGGATCCATGTGTGGTAACACCATGTC *(BamHI)*	pcDNA3.1-*APT2-3Flag*/co-IP
*APT2-3Flag-*R	TGCTGGATATCTGCAGAATTCTCACTTATCGTCGTCATCCTTGTAATCGATCTTATCGTCGTCATCCTTGTAATCTCCCTTATCGTCGTCATCCTTGTAATCGACAGGAGGCAGCAGCTTCT *(EcoRI)*	

^a^Sequences of restriction sites are underline

**Fig. 1:**
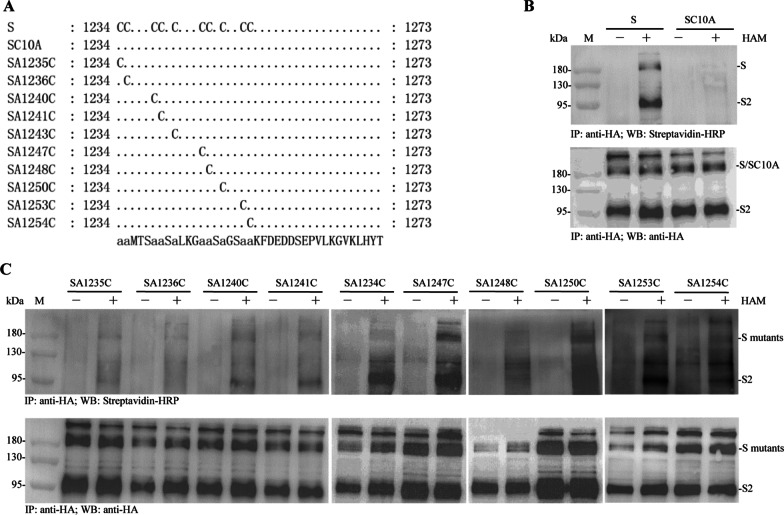
The palmitoylation levels of S protein and its mutants measured by ABE assays. **a** Multiple amino acid sequence alignments of the endodomains (1234–1273 aa) of SARS-CoV-2 S protein and its eleven mutants. The dark spots indicate amino acids on the last line. The small letter “a” indicates that the cysteine residue was replaced with an alanine residue. SC10A represents that all ten cysteine residues in the endodomain were changed to alanine residues. SA1235C ~ SA1254C represent that only one cysteine residue in the endodomain was reserved and the other nine cysteine residues were changed to alanine residues. **b** The palmitoylation levels of S protein and its mutant SC10A. HEK293T cells were transfected with plasmid expressing S-HA or SC10A-HA for 48 h. The palmitoylation levels of S and SC10A were measured by ABE assays and the cells were then immunoblotted with streptavidin-HRP and anti-HA antibody. M: protein molecular mass marker. **C** The palmitoylation levels of ten mutants SA1235C ~ SA1254C. HEK293T cells were transfected with ten mutant plasmids expressing SA1235C ~ SA1254C for 48 h. The palmitoylation levels of the ten mutants were measured by ABE assays

To generate the plasmids for analysis of palmitoylation levels of SARS-CoV-2 S protein and its mutant SC10A which represents that all ten cysteine residues in the endodomain (1234–1273 aa) were changed to alanine residues (Fig. 1a), the open reading frame (ORF) of SARS-CoV-2 *S* gene was amplified using pcDNA3.1-*S* plasmid as template with primers *S*-F2/*S*-*HA*-R and cloned into pcDNA3.1(+) to produce pcDNA3.1-*S*-*HA*. The mutant SC10A was amplified using the plasmid pEGFP-*SC10A* described below as a template with primers *S*-F2/*S*-*HA*-R and cloned into pcDNA3.1(+) to produce pcDNA3.1-*SC10A*-*HA*.

To generate plasmids for identification of the palmitoylated cysteine residues of SARS-CoV-2 S protein, ten mutants SA1235C ~ SA1254C (in which only one cysteine residue in the endodomain was reserved and the other nine cysteine residues were changed to alanine residues) were constructed (Fig. 1a). For example, two fragments SA1235C-N and SA1235C-C were amplified from pcDNA3.1-*SC10A*-*HA* plasmid with primers *S*-F2/*SA1235C*-R and *SA1235C*-F/*S*-*HA*-R, respectively. The fragment SA1235C was amplified via overlap PCR using SA1235C-N and SA1235C-C as templates and cloned into plasmid pcDNA3.1(+) to produce pcDNA3.1-*SA1235C*-*HA*. As described above, primers *S*-F2/*SA1236C*-R and *SA1236C*-F/*S*-*HA*-R were used to construct the plasmid pcDNA3.1-*SA1236C*-*HA*. Similarly, eight other plasmids expressing S protein mutants were constructed.

To generate the plasmids for co-IP assays, the ORFs of the *ZDHHC5* and *GOLGA7* genes were amplified using HEK293T cDNA as templates with primers *ZDHHC5*-F/*ZDHHC5*-*3Flag*-R and *GOLGA7*-F/*GOLGA7*-*3Flag*-R, respectively. To generate a plasmid expressing the mutant ZDHHC5-C134S (cysteine residue 134 in the DHHC motif of ZDHHC5 was changed to a serine residue). Two fragments ZDHHC5-C134S-N and ZDHHC5-C134S-C were amplified with primers *ZDHHC5*-F/*ZDHHC5*-*C134S*-R and *ZDHHC5*-*C134S*-F/*ZDHHC5*-*3Flag*-R, respectively. The fragment ZDHHC5-C134S was amplified via overlap PCR using ZDHHC5-C134S-N and ZDHHC5-C134S-C as templates. ZDHHC5△PDZ representing ZDHHC5 lacking a PDZ-binding domain (aa 711 ~ 715, YEISV) was amplified with primers *ZDHHC5*-F/*ZDHHC5△PDZ*-*3Flag*-R. The ORFs of *APT1*/*APT2* genes were amplified using HEK293T cDNA as templates with primers *APT1*-F/*APT1*-*3Flag*-R and *APT2*-F/*APT2*-*3Flag*-R, respectively. All six fragments obtained above were separately cloned into pcDNA3.1(+) to construct the corresponding plasmids.

Two plasmids expressing S protein fused with enhanced green fluorescent protein (S-EGFP) or SC10A fused with EGFP (SC10A-EGFP) were constructed to analyze their subcellular localizations. The ORFs of SARS-CoV-2 *S* gene and the mutant SC10A were amplified with primers *S*-F/R and *S*-F/*SC10A*-R and then cloned into pEGFP-N3 to produce pEGFP-*S* and pEGFP-*SC10A*, respectively. Four plasmids expressing ZDHHC5, ZDHHC5-C134S, ZDHHC5△PDZ and GOLGA7 separately fused with red fluorescent protein (RFP) were constructed to analyze their subcellular localizations. The ORF of the *ZDHHC5* or *GOLGA7* gene was separately amplified with primers *ZDHHC5*-F2/R2 or *GOLGA7*-F2/R2. The fragment ZDHHC5-C134S was amplified with primers *ZDHHC5*-F2/R2 using pcDNA3.1-*ZDHHC5*-*C134S* as template. The fragment ZDHHC5△PDZ was amplified with primers *ZDHHC5*-F2/*ZDHHC5△PDZ*-R using pcDNA3.1-*ZDHHC5*-*3Flag* as template. The four fragments obtained above were separately cloned into pDsRed2-C1 to produce the corresponding plasmids.

Two pairs of oligos *ZDHHC5*-*sgRNA*-F/R and *GOLGA7*-*sgRNA*-F/R were designed according to a previous report [15]. The fragments ZDHHC5-sgRNA and GOLGA7-sgRNA were obtained by annealing and separately cloned into plasmid lentiCRISPRv2. The plasmids pMD2-G and psPAX2 were purchased from Addgene (www.addgene.org).

### ABE assays

The ABE assays were performed as previously described with minor modifications [17, 19]. In brief, HEK293T, HEK293T-ZDHHC5^KO^ or HEK293T-GOLGA7^KO^ cells transiently expressing HA-tagged S or its mutants SC10A and SA1235C ~ SA1254C were harvested at 48 h post transfection (hpt) and washed with cold phosphate-buffered saline (PBS). Prior to cell lysis, N-ethylmaleimide (NEM) was dissolved in 100% EtOH and added to the Lysis Buffer (50 mM Tris–HCl pH 7.5, 150 mM NaCl, 1% IGEPAL CA-630, 10% glycerol) with 1 mM phenylmethylsulfonyl fluoride (PMSF) and protease inhibitor cocktail (Sigma) to a final concentration of 50 mM. Cells were then suspended in NEM containing Lysis buffer for 1 h at 4 °C and the supernatants were incubated with Red Anti-HA Affinity Gel (Sigma) overnight at 4 °C. After incubation, the beads were washed one time with Lysis Buffer with 10 mM NEM, one time with Stringent Buffer (Lysis Buffer with 10 mM NEM and 0.1% SDS), and then three times with Lysis Buffer of pH 7.2. Then, beads were incubated with a freshly prepared hydroxylamine (HAM)-containing Lysis Buffer of pH 7.2 with 1 M HAM at room temperature for 1 h and washed four times with Lysis Buffer of pH 7.2 and three times with Lysis Buffer of pH 6.2. Subsequently, beads were treated with Biotin-BMCC (5 μM) in Lysis Buffer of pH 6.2 at 4 °C for 1 h. The immunoprecipitated samples were analyzed by Western blot analysis using anti-HA mouse monoclonal antibody (1:1000, Cell Signaling) and streptavidin-HRP (1:1250).

### Co-IP assays

**Fig. 2:**
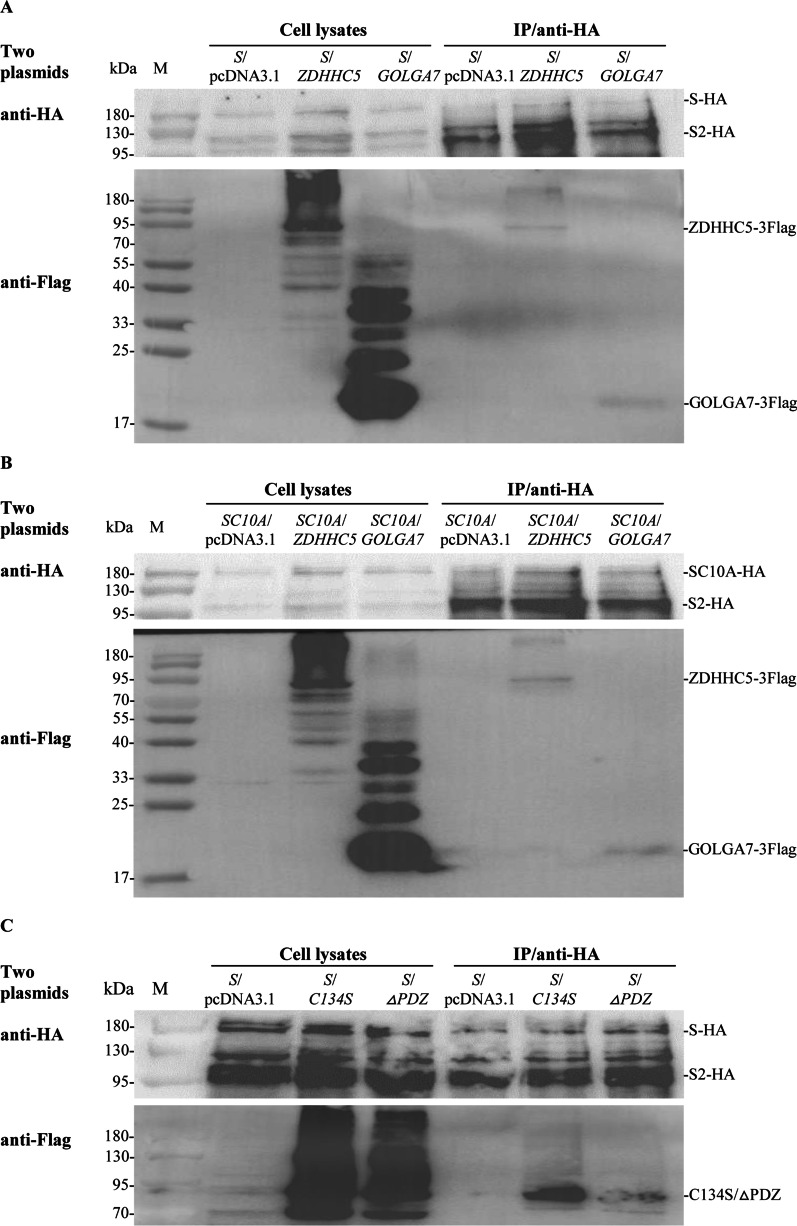
Analyzing interactions between SARS-CoV-2 S, SC10A and ZDHHC5, its mutants or GOLGA7 by co-IP. **a** Interaction between SARS-CoV-2 S protein and ZDHHC5 or GOLGA7. Cell lysates and immunoprecipitated protein complexes (IP) from HEK293T cells cotransfected with indicated plasmids *S*/*pcDNA3.1*, *S*/*ZDHHC5* and *S*/*GOLGA7* were subjected to Western blot analysis using anti-HA and anti-Flag antibodies. Cells lysates and IP showed the bands of S-HA, S2-HA, ZDHHC5-3Flag (81 kDa) and GOLGA7-3Flag (19 kDa). **b** Interaction between SC10A and ZDHHC5 or GOLGA7. Cell lysates and IP from HEK293T cells cotransfected with the indicated plasmids (*SC10A*/*pcDNA3.1*, *SC10A*/*ZDHHC5* and *SC10A*/*GOLGA7*) were subjected to Western blot analysis. **c** Interaction between S protein and ZDHHC5-C134S (C134S) or ZDHHC5△PDZ (△PDZ) by co-IP. Cell lysates and IP from HEK293T cells cotransfected with the indicated plasmids (*S*/*pcDNA3.1*, *S*/*C134S* and *S*/*△PDZ*) were subjected to Western blot analysis

Co-IP assays were performed as previously described with minor modifications [20]. Briefly, HEK293T cells seeded in 10 cm dishes were cotransfected with 7.5 μg of pcDNA3.1-*S*-*HA* and 7.5 μg of pcDNA3.1-*ZDHHC5*-*3Flag* or other indicated plasmids. As a control, 7.5 μg of empty vector pcDNA3.1(+) was transfected in parallel. At 24 hpt, cells were lysed with 1 mL of a radio immunoprecipitation assay (RIPA) buffer (50 mM Tris pH 7.4, 150 mM NaCl, 1% NP-40, 0.25% sodium deoxycholate) containing protease inhibitor cocktail (Sigma) and 1 mM PMSF for 1 h. The cell lysates were centrifuged to remove cell debris and the lysate supernatants were collected and divided into two parts. Five hundred microliters of lysate supernatant was used to analyze the co-expression of S protein and other proteins by Western blot analysis using anti-HA and anti-Flag antibodies. Other 500 μL of lysate supernatant was incubated with Red Anti-HA Affinity Gel (Sigma) overnight at 4 °C. The precipitates were collected by centrifugation, washed with ice-cold PBS and RIPA buffer, eluted with 40 μL PBS, and finally subjected to Western blot analysis. Since the band of S protein in cell supernatants was usually very weak by Western blot analysis using anti-HA antibody as shown in Fig. 2a, 4b, 440 μL of lysate supernatants were used to enrich S protein with 25 μL of Red Anti-HA Affinity Gel and other 60 μL of lysate supernatants were used to analyze the expression of other proteins when the interaction between S protein and ZDHHC5, ZDHHC5-C134S, ZDHHC5△PDZ, APT1 or APT2 was detected (Fig. 2c, 4a, 7).

### Western blot analysis

Western blot analysis was performed as described previously [20]. Protein samples were resolved by SDS-PAGE, followed by electroblotting to polyvinylidene difluoride (PVDF) membranes. The blots were probed with anti-HA mouse monoclonal antibody (1:1000, Cell Signaling), anti-Flag rabbit monoclonal antibody (1:1000, Cell Signaling), ZDHHC5-specific rabbit polyclonal antiserum (1:100, Sigma), and GOLGA7-specific rabbit polyclonal antiserum (1:1000, Abclonal). Peroxidase-conjugated goat anti-mouse or anti-rabbit IgG (H + L) antibody was used as the secondary antibody. The signals were detected with a chemiluminescent horseradish peroxidase (HRP) substrate (Millipore).

### CRISPR/Cas9 gene editing

CRISPR/Cas9 gene editing was performed as previously described with minor modifications [15]. HEK293T, A549 and Hela cells lacking ZDHHC5 or GOLGA7 were generated as follows. Approximately 8 × 10^5^ HEK293T cells in six-well plates were cotransfected with 0.51 μg of pMD2-G, 0.78 μg of psPAX2 and 1.20 μg of lentiCRISPRv2-*ZDHHC5 sgRNA* or lentiCRISPRv2-*GOLGA7 sgRNA*. At 48 hpt and 72 hpt, the supernatants were collected and mixed. HEK293T, A549 or Hela cells in six-well plates were inoculated with 500 μL of harvested supernatant for 24 h and then selected with DMEM supplemented with 10% FBS containing 0.9 μg/mL (for HEK293T cells), 2 μg/mL (for A549 cells) or 1 μg/mL (for Hela cells) puromycin until clones formed. All clones were sorted into 96-well plates by flow cytometer (FACSAria SORP) and each well contained only one cell. Individual clones formed in 96-well plates were further transferred into 6-well plates for culture and then identified by DNA sequencing and Western blot analysis. For DNA sequencing, the genomes of cells in 6-well plates were extracted and amplified by PCR using *ZDHHC5*-F3/R3 or *GOGLA7*-F3/R3 primers. The PCR products were sequenced using *ZDHHC5*-seq-F or *GOLGA7*-seq-F primer.

### Fluorescence microscopy

Hela or A549 cells seeded on the surface of coverslips placed in 6-well plates were alone transfected with plasmid pEGFP-N3, pEGFP-*S*, pEGFP*-SC10A*, pDsRed2-C1, pDsRed2-*ZDHHC5*, pDsRed2-*ZDHHC5-C134S,* pDsRed2-*ZDHHC5△PDZ*, pDsRed2-*GOLGA7* or cotransfected with plasmids pEGFP-*S* + pDsRed2-*ZDHHC5*/*GOLGA7*, pEGFP-*SC10A* + pDsRed2-*ZDHHC5*/*GOLGA7*. At 24 hpt, cells were fixed with 4% paraformaldehyde, permeabilized with 0.2% Triton X-100, stained by Hoechst 33,342 and observed under a Leica DMi8 fluorescence microscope (objective 64 ×) or a Leica Stellaris laser confocal microscope (objective 100 ×), as described previously [21].

### Immunofluorescence assay (IFA)

To further confirm the subcellular localization of SARS-CoV-2 S protein, an immunofluorescence assay was performed as previously described with minor modifications [22]. Hela cells transfected with plasmid pcDNA3.1-*S-HA* for 24 h were fixed with 4% paraformaldehyde for 30 min, permeabilized with 0.2% Triton X-100 and blocked in 10% bovine serum albumin (BSA) at room temperature for 1 h. The cells were incubated with anti-HA mouse monoclonal antibody (1:100) in 1% BSA for 2 h, rinsed three times for 10 min each with PBS containing 1% BSA, and then incubated with Alexa-488-conjugated goat anti-mouse IgG (1:500, Invitrogen). The cells were stained by Hoechst 33,342 and examined under a Leica Stellaris laser confocal microscope (objective 100 ×).

### SARS-CoV-2 spike-mediated pseudovirus entry assay

To analyze the effects of ZDHHC5 or GOLGA7 knockout on SARS-CoV-2 pseudovirus entry, 5 × 10^4^ A549, A549-ZDHHC5^KO^, A549-GOLGA7^KO^, Hela, Hela-ZDHHC5^KO^ or Hela-GOLGA7^KO^ cells were seeded in 96-well white plates and grown overnight. The culture medium was replaced with fresh medium containing 8 μg/mL polybrene for 1 h and then the cells were inoculated with 4E + 6 RLU (relative light units) VSV-SARS-2-S-luc pseudovirus purchased from Delivectory Biosciences Inc. At 16 h post infection (hpi), the culture medium was replaced with fresh medium. Entry efficiency was quantified at 48 hpi by measuring the activity of *Renilla* luciferase in cell lysates using the ONE-Glo™ Luciferase Assay (E6120, Promega, USA) according to the manufacturer’s instructions (PekinElmer Envision). The infection experiments were performed under biosafety level 2 (BSL2) laboratory conditions.

## Results

### Identification of palmitoylated cysteine residues of SARS-CoV-2 S protein

The palmitoylation levels of SARS-CoV-2 S protein and its eleven mutants SC10A, SA1235C ~ SA1254C (Fig. 1a) were analyzed by ABE assays. Ectopically expressed S protein and S2 subunit were obviously palmitoylated, but SC10A was not palmitoylated. Loss of signal upon omission of HAM treatment demonstrated that S protein incorporates palmitate through a thioester linkage (Fig. 1b). All ten mutants SA1235C ~ SA1254C were palmitoylated, although the palmitoylation levels of the two mutants SA1236C and SA1248C or their S2 subunits were very weak (Fig. 1c).

### Confirmation of interaction between SARS-CoV-2 S protein and ZDHHC5 or GOLGA7 by co-IP

The interaction between SARS-CoV-2 S protein and ZDHHC5 or GOLGA7 was analyzed by co-IP assays. S protein and ZDHHC5 or GOLGA7 were coexpressed in HEK293T cells by cotransfection with indicated plasmids (*S*/*ZDHHC5* and *S*/*GOLGA7*). The cell lysates and immunoprecipitated protein complexes (IP) were detected by Western blot analysis using anti-HA and anti-Flag antibodies. As the results, the bands of S protein and S2 subunit were detected in both cell lysates and IP by anti-HA, which were weak in cell lysates and strong in IP (Fig. 2a, anti-HA). This suggested that the fusion protein S-HA and S2 subunit were effectively immunoprecipitated by anti-HA affinity agarose gel. The bands of ZDHHC5-3Flag (81 kDa) and GOLGA7-3Flag (19 kDa) were detected in both cell lysates and IP by anti-Flag. In addition, a band of more than 180 kDa was observed when ZDHHC5-Flag was detected in both cell lysates and IP (Fig. 2a, anti-Flag). As a negative control (*S*/*pcDNA3.1*), only S-HA and S2 subunit were detected in cell lysates and IP. These further confirmed that SARS-CoV-2 S protein interacted with ZDHHC5 or GOLGA7.

The effects of the ten cysteine residues in the endodomain of S protein on the interaction between S protein and ZDHHC5 or GOLGA7 were analyzed by co-IP. SC10A and ZDHHC5 or GOLGA7 were coexpressed in HEK293T cells by cotransfection with indicated plasmids (*SC10A*/*ZDHHC5* and *SC10A*/*GOLGA7*). The bands of SC10A and its S2 subunit were detected in both cell lysates and IP by anti-HA (Fig. 2b, anti-HA). The bands of ZDHHC5-3Flag and GOLGA7-3Flag were detected in both cell lysates and IP by anti-Flag (Fig. 2b, anti-Flag). These showed that SARS-CoV-2 SC10A interacted with ZDHHC5 or GOLGA7.

The interactions between S protein and the two mutants of ZDHHC5 including ZDHHC5-C134S or ZDHHC5△PDZ were further analyzed by co-IP. The results showed that S protein still interacted with the two mutants (Fig. 2c).

### Construction of three cell lines lacking ZDHHC5 or GOLGA7 by CRISPR/Cas9

**Fig. 3:**
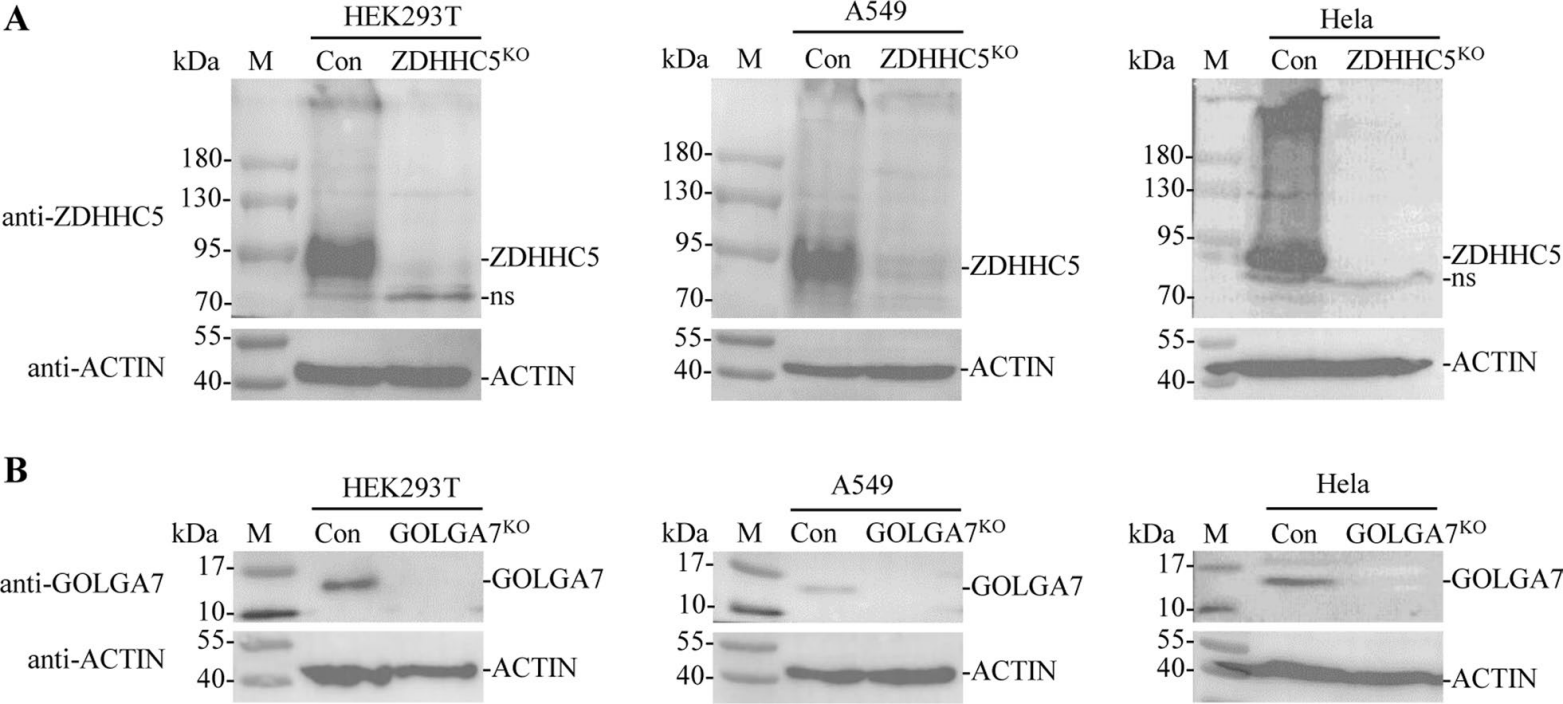
Validation of *ZDHHC5* or *GOLGA7* gene editing by CRISPR-Cas9. **a** ZDHHC5 or **b** GOLGA7 expression in gene-disrupted HEK293T, A549 and Hela cells was determined by Western blot analysis using antibody anti-ZDHHC5 or anti-GOLGA7. ns indicates non-specific band

The *ZDHHC5* or *GOLGA7* gene in three cell lines HEK293T, A549 and Hela was edited by CRISPR/Cas9 to investigate the effects of ZDHHC5 or GOLGA7 knockout on S protein’s subcellular localization, palmitoylation levels and pseudovirus entry. *ZDHHC5* or *GOLGA7* gene editing was first verified by PCR amplification and DNA sequencing. The DNA fragments were amplified from the genomes of edited cells with primers *ZDHHC5*-F3/R3 or *GOLGA7*-F3/R3 and then sequenced, indicating that *ZDHHC5* or *GOLGA7* gene was edited in all three cell lines. Western blot analysis showed that a specific band of about 78 kDa or 16 kDa was detected in the control cells, but not in the edited cells (Fig. 3a, b), confirming that the expression of ZDHHC5 or GOLGA7 was completely interrupted in the three cell lines.

### The interaction between S protein and ZDHHC5 or GOLGA7 in HEK293T cells lacking the other

**Fig. 4:**
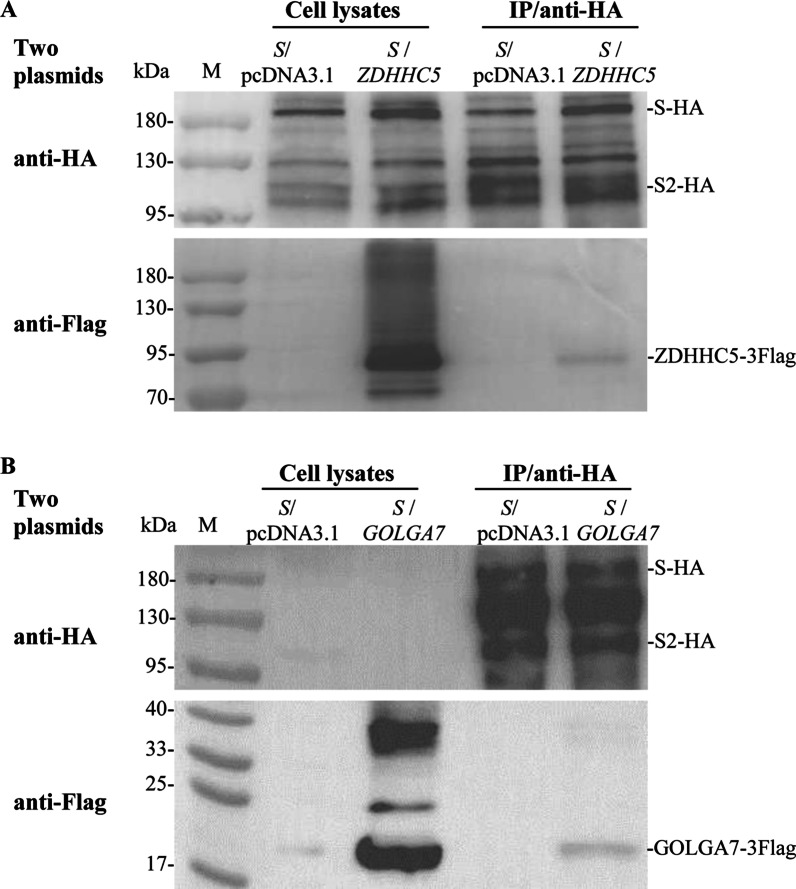
Analyzing interactions between SARS-CoV-2 S and ZDHHC5/GOLGA7 in HEK293T cells lacking one protein by co-IP. **a** Interaction between S protein and ZDHHC5 in HEK293T-GOLGA7^KO^ cells. **b** Interaction between S protein and GOLGA7 in HEK293T-ZDHHC5^KO^ cells

The interaction between S protein and ZDHHC5 in HEK293T-GOLGA7^KO^ cells and the interaction between S protein and GOLGA7 in HEK293T-ZDHHC5^KO^ cells were further analyzed by co-IP, respectively. The results demonstrated that S protein still interacted with ZDHHC5 in HEK293T-GOLGA7^KO^ cells (Fig. 4a) and with GOLGA7 in HEK293T-ZDHHC5^KO^ cells (Fig. 4b).

### Localizations of SARS-CoV-2 S protein, ZDHHC5 and GOLGA7

**Fig. 5:**
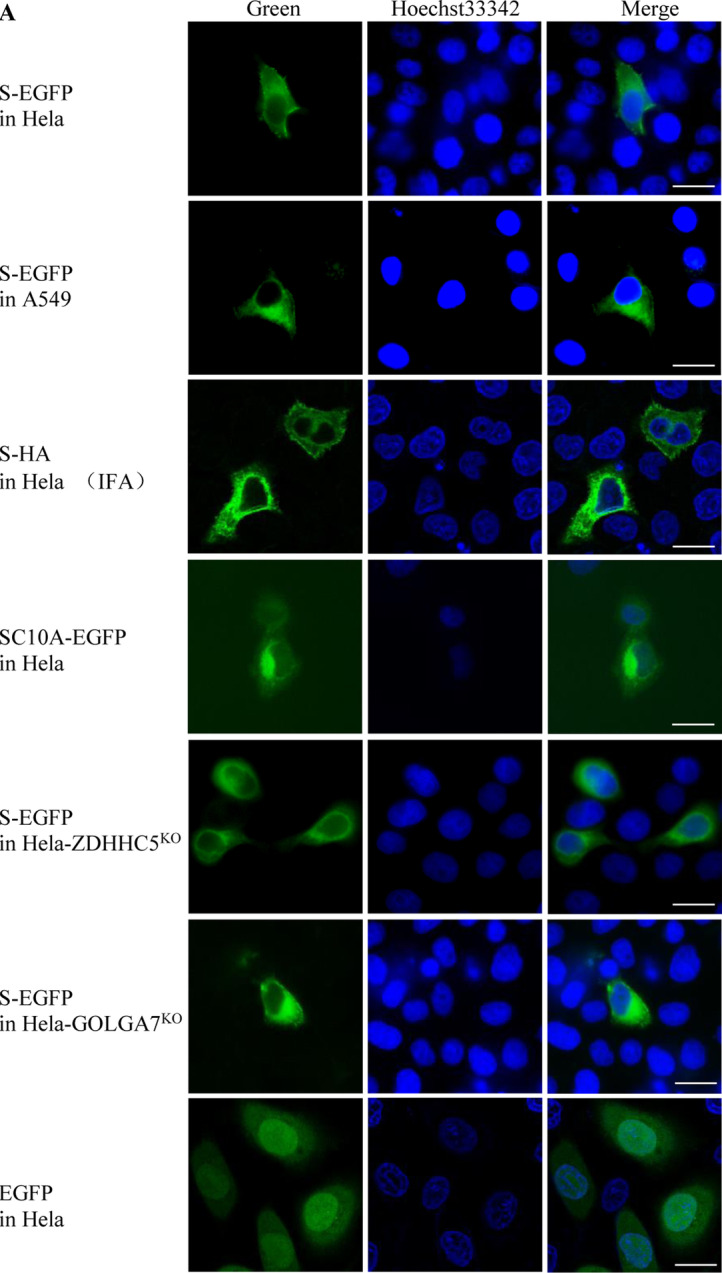

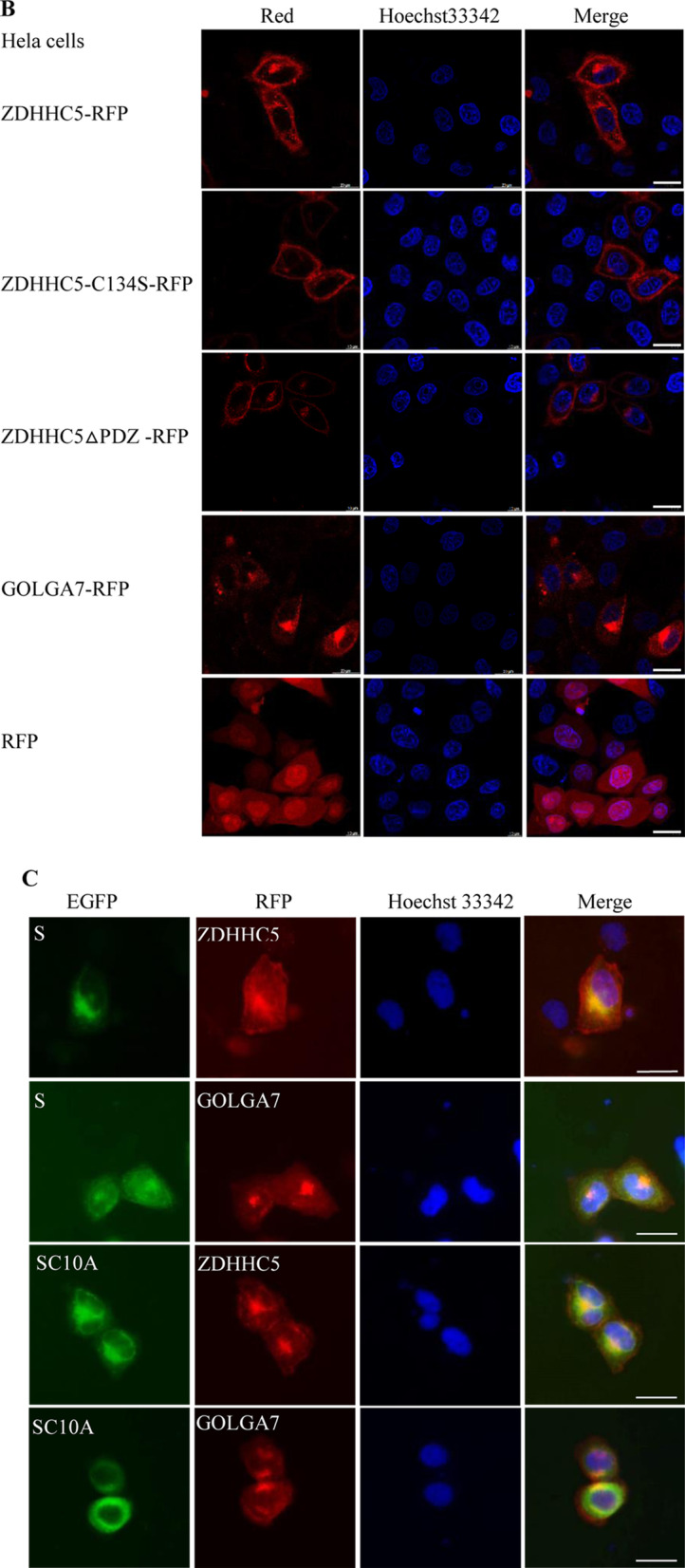
Fluorescence micrographs of cells expressing a single protein or co-expressing two proteins. **a** Expressing protein S-EGFP, S-HA or SC10A-EGFP alone in Hela, A549, Hela-ZDHHC5^KO^ or Hela-GOLGA7^KO^ cells. Scale bar: 20 μm. **b** Expressing protein ZDHHC5-RFP, ZDHHC5-C134S-RFP, ZDHHC5△PDZ-RFP or GOLGA7-RFP alone in Hela cells. Scale bar: 20 μm. **c** Co-expressing two proteins, S-EGFP + ZDHHC5-RFP/GOLGA7-RFP and SC10A-EGFP + ZDHHC5-RFP/GOLGA7-RFP in Hela cells, respectively. S-EGFP (green), SC10A-EGFP (green), ZDHHC5-RFP (red), GOLGA7-RFP (red), nucleus (blue), and colocalization (yellow). Scale bar: 20 μm

Fluorescence microscopy showed that S-EGFP distributed in the cytoplasm of Hela and A549 cells, although a weak fluorescence signal was observed in the plasma membrane of some cells. An immunofluorescence assay (IFA) showed that S-HA distributed in the cytoplasm of Hela cells. SC10A-EGFP also distributed in the cytoplasm of Hela. S-EGFP also distributed in the cytoplasm of Hela-ZDHHC5^KO^ and Hela-GOLGA7^KO^ cells (Fig. 5a). As a control, the EGFP distributed in both the cytoplasm and the nucleus of Hela cells.

ZDHHC5-RFP, ZDHHC5-C134S-RFP, ZDHHC5△PDZ-RFP all distributed in the cytoplasm and plasma membrane of Hela cells. GOLGA7 mainly distributed in the cytoplasm and little distributed in the plasma membrane (Fig. 5b). As a control, the RFP distributed in both the cytoplasm and the nucleus of Hela cells.

When Hela cells were cotransfected, S-EGFP/SC10A-EGFP colocalized with ZDHHC5-RFP/GOLGA7-RFP in the cytoplasm, respectively (Fig. 5c). These not only further confirmed the interaction of S protein with ZDHHC5 or GOLGA7, but also indicated that the palmitoylation of S protein had no significant effect on their colocalization.

### The effects of ZDHHC5 or GOLGA7 knockout on the palmitoylation levels of SARS-CoV-2 S protein and pseudovirus entry

**Fig. 6:**
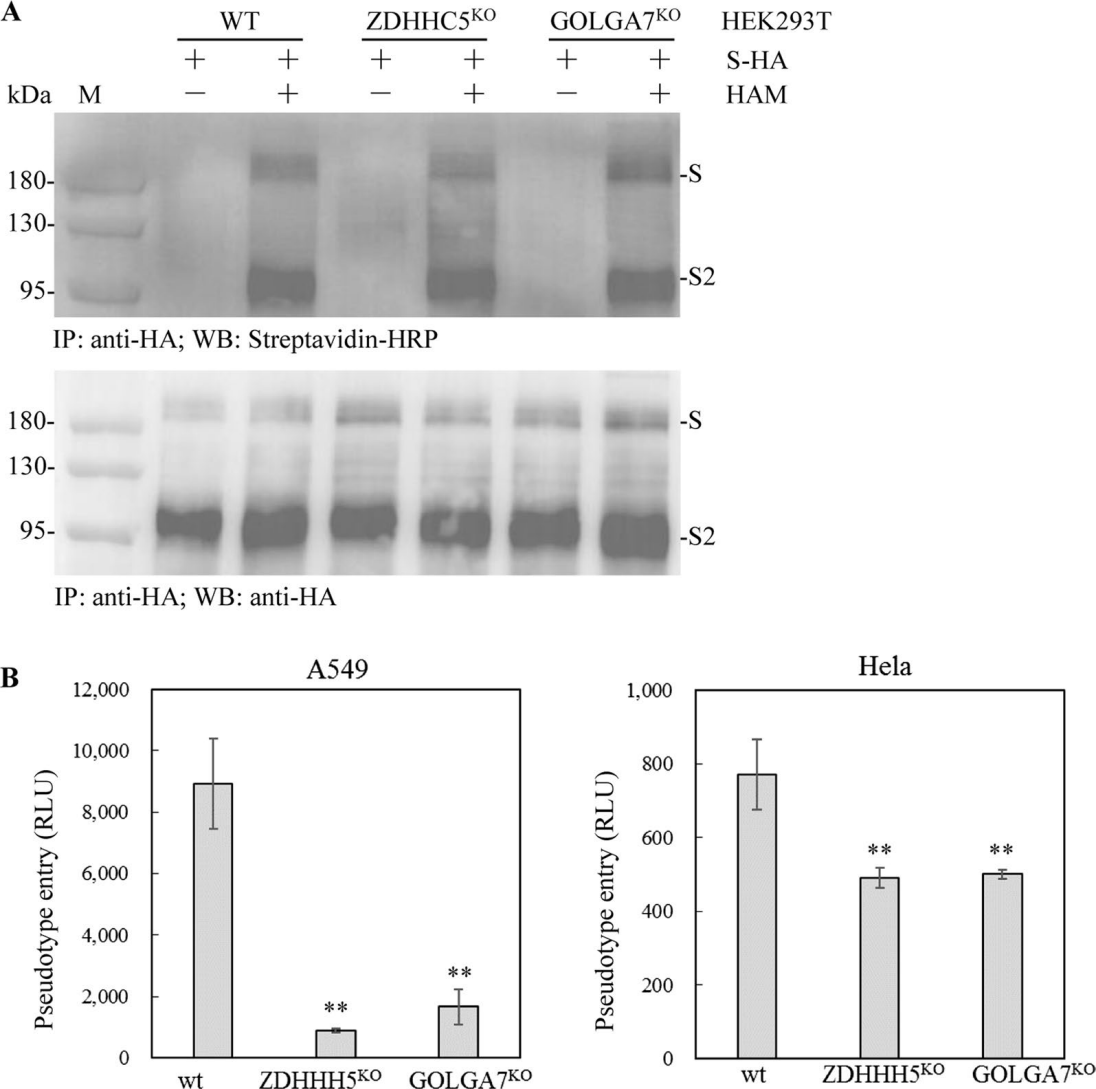
The effects of ZDHHC5 or GOLGA7 knockout on S protein palmitoylation and pseudovirus entry. **a** HEK293T, HEK293T-GOLGA7^KO^ and HEK293T-ZDHHC5^KO^ cells were transfected with plasmid expressing S-HA for 48 h. The palmitoylation levels of S protein were measured by ABE assays. **b** Cells were infected with an equal number of SARS-CoV-2 pseudotyped virions and the entry efficiencies were quantified at 48 hpi by measuring luciferase activity (in relative light units, RLU) (n = 3). ZDHHC5 or GOLGA7 knockout in A549 and Hela cells significantly reduced pseudovirus infection. Error bars indicate standard deviations. ** represents P < 0.01

The effect of ZDHHC5 or GOLGA7 knockout on the palmitoylation levels of S protein was analyzed by ABE assays. Ectopically expressed S protein and S2 subunit were palmitoylated in HEK293T, HEK293T-ZDHHC5^KO^ and HEK293T-GOLGA7^KO^ cells and the palmitoylation levels were comparable (Fig. 6a).

The effects of ZDHHC5 or GOLGA7 knockout on SARS-CoV-2 pseudovirus entry into Hela and A549 cells were further analyzed. The entry efficiency of SARS-CoV-2 pseudovirus into A549 cells was significantly higher than that into A549-ZDHHC5^KO^ (*P* = 0.0007) and A549-GOLGA7^KO^ (*P* = 0.0013) cells. Similarly, the entry efficiency of SARS-CoV-2 pseudovirus into Hela cells was significantly higher than that into Hela-ZDHHC5^KO^ (*P* = 0.0084) and Hela-GOLGA7^KO^ (*P* = 0.0086) cells (Fig. 6b).

Furthermore, the entry efficiency of SARS-CoV-2 pseudovirus into A549 cells was significantly higher than that into Hela cells by approximately 11.5-fold (*P* = 0.0007).

### Interaction between SARS-CoV-2 S protein and APT2 determined by co-IP

**Fig. 7:**
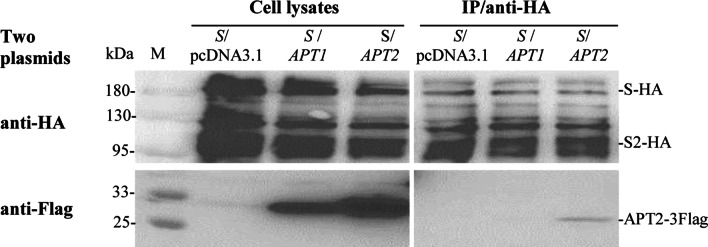
Analyzing interactions between SARS-CoV-2 S protein and APT1/APT2 by co-IP. Cell lysates and IP from HEK293T cells cotransfected with indicated plasmids *S*/*pcDNA3.1*, *S*/*APT1* and *S*/*APT2* were subjected to Western blot analysis using anti-HA and anti-Flag antibodies

The interactions between S protein and two depalmitoylases APT1/APT2 were also analyzed by co-IP assays. The results demonstrated that S protein interacted with APT2, but not APT1 (Fig. 7).

## Discussion

The aims of this study were to identify the palmitoylated cysteine residues of S protein, investigate the effects of ZDHHC5 or GOLGA7 knockout on S protein’s subcellular localization, palmitoylation, pseudovirus entry and identify the enzyme for depalmitoylation of S protein. The main results showed that ten cysteine residues in the endodomain of SARS-CoV-2 S protein were palmitoylated. The interaction and colocalization of S protein with ZDHHC5 or GOLGA7 were independent of the ten cysteine residues in the endodomain of S protein. ZDHHC5 or GOLGA7 knockout had no significant effect on S protein’s subcellular localization or palmitoylation, but significantly decreased the entry efficiencies of SARS-CoV-2 pseudovirus into A549 and Hela cells. Moreover, the S protein interacted with the depalmitoylase APT2.

S proteins of several coronaviruses have been shown to be palmitoylated. For example, palmitoylation of SARS-CoV S protein is important for spike-mediated cell fusion and is necessary for partitioning into detergent-resistant membranes [7, 23]. The palmitoylation of S protein from murine coronavirus is essential for virion assembly and infectivity and is important for interaction with the M protein [8]. SARS-CoV-2 S protein undergoes palmitoylation and alteration of the ten cysteine residues in the endodomain of S protein decreases the efficiency of syncytium formation, cell–cell fusion and pseudotyped SARS-CoV-2 infectivity [6], but the exact palmitoylated cysteine residues are not yet known. Here, ABE assays showed that ten cysteine residues in the endodomain of S protein were palmitoylated, although the palmitoylation levels of the two mutants SA1236C and SA1248C or their S2 subunits were very weak. It has been reported that the replacement of each of four cysteine clusters in the endodomain of SARS-CoV-2 S protein by two or three alanine residues decreased the palmitoylation levels to different degrees [7]. It is possible that different cysteine residues in the endodomain of SARS-CoV-2 S protein have differential rates of palmitate turnover.

The complex ZDHHC5/GOGAL7 that interacts with S protein was first detected by AP-MS [14]. Here, the interaction of S protein with ZDHHC5 or GOLGA7 was confirmed by co-IP. The mutant SC10A interacted and colocalized with ZDHHC5 or GOLGA7, suggesting that the interaction and colocalization of S protein with ZDHHC5 or GOLGA7 were independent of the ten cysteine residues in the endodomain of S protein. S protein still interacted with the two mutants of ZDHHC5 including ZDHHC5-C134S or ZDHHC5△PDZ, indicating that the interaction between S protein and ZDHHC5 was independent of the enzymatic activity and the PDZ-binding domain of ZDHHC5. ZDHHC5 usually interacts with substrate proteins through its own PDZ binding domain and its DHHC motif also affects its interactions with substrate proteins [17, 18]. These implied that ZDHHC5 might interact with different substrate proteins via different binding sites. A band of more than 180 kDa was observed when ZDHHC5 was detected by co-IP. The band should be the trimer of ZDHHC5, since it can form monomer and aggregation form of higher molecular mass [16].

Fluorescence microscopy showed that S-EGFP distributed in the cytoplasm of Hela and A549 cells. S-HA also distributed in the cytoplasm of Hela cells. Obviously, the localizations of S protein in the two kinds of cells from different tissues were consistent. SARS-CoV-2 S protein was distributed in the cytoplasm of Human Epithelial-2 (HEp-2) cells in a previous report [24]. SC10A-EGFP also distributed in the cytoplasm of Hela, indicating that alteration of the ten cysteine residues in the endodomain had no significant effect on the subcellular localization of S protein. S-EGFP also distributed in the cytoplasm of Hela-ZDHHC5^KO^ and Hela-GOLGA7^KO^ cells, revealing that ZDHHC5 or GOLGA7 knockout had no significant effect on the subcellular localization of S protein. ZDHHC5-RFP, ZDHHC5-C134S-RFP, ZDHHC5△PDZ-RFP all distributed in the cytoplasm and plasma membrane of Hela cells, indicating that the enzymatic activity and PDZ-binding domain of ZDHHC5 had no significant effect on its subcellular localization.

In this study, ZDHHC5 knockout had no significant effects on the subcellular localization or palmitoylation levels of S protein, but significantly decreased the entry efficiencies of SARS-CoV-2 pseudovirus into A549 and Hela cells. These implied that one or more other palmitoyltransferases might be involved in the palmitoylation of S protein. During the preparation of this manuscript, Mesquita et al. reported that ZDHHC5 knockdown in Hela cells or ZDHHC5 knockout in HAP-1 (human astrocyte precursor) cells had no significant effect on the palmitoylation of ectopically expressed S protein, but ZDHHC5 knockdown in Vero E6 (African green monkey kidney cells) significantly decreased S protein palmitoylation during SARS-CoV-2 infection [25]. Moreover, S protein palmitoylation was mainly mediated by ZDHHC8,9,20 via analyzing spike-incorporated radioactivity in Hela cells cotransfected with individual siRNAs targeting all human ZDHHCs [25], while Puthenveetil et al. reported that ZDHHC2,3,6,11,20,21,24 were as putative palmitoylation enzymes for SARS-CoV-2 S protein modification by click-chemistry-based analyses of coexpression of S protein with individual ZDHHC [26]. Our results are consistent with the report of Mesquita et al. However, in other reports, the interactions between SARS-CoV-2 S protein and ZDHHC5/GOLGA7 were confirmed by co-IP and the two host protein overexpressions in HEK293T cells enhanced S protein palmitoylation synergistically and ZDHHC5 knockdown decreased S protein palmitoylation and pseudovirus infection [27, 28]. All these distinct results were likely caused by different analytic systems, such as cell lines and detection methods. Therefore, how ZDHHC5 impacts the palmitoylation of the S protein for virus entry needs more investigation.

Why did ZDHHC5 knockout generate the two different outcomes in pseudovirus entry? Probably because ZDHHC5 knockout could affect the palmitoylation of S protein at earlier infected stage, which led to the decrease of the pseudovirus entry efficiency, suggesting that the palmitoylation of S protein at later infected stages is catalyzed by other palmitoyltransferase members. Moreover, the ZDHHC5 knockout likely affects the functions of other cognate substrates, such as nucleotide oligomerization domain (NOD)-like receptors 1 and 2 (NOD1/2) which are involved in infecting of SARS-CoV-2, to decrease the entry efficiency of pseudovirus [17, 18]. As previously reported, NOD1 is required for recognition of SARS-CoV-2 in lung epithelial cells [29]. A drug that targets NOD2 was shown to have potent broad-spectrum antiviral activity against several viruses, including SARS-CoV-2 [30]. However, more investigation are required to address above mentioned questions.

In addition to ZDHHC5, the role of the accessory protein GOLGA7 was also investigated. S protein interacted with ZDHHC5 in HEK293T-GOLGA7^KO^ cells and with GOLGA7 in HEK293T-ZDHHC5^KO^ cells. GOLGA7 knockout had no significant effects on the subcellular localization or palmitoylation levels of S protein, but significantly decreased the entry efficiencies of SARS-CoV-2 pseudovirus into A549 and Hela cells. It has been reported that GOLGA7 interacts with ZDHHC8,9, and regulates the enzyme activity and stability of ZDHHC9 [12, 31]. ZDHHC8,9 were involved in the palmitoylation of S protein in a recent report [25]. These implied that GOLGA7 knockout might impair the enzymatic activity and disrupt the stability of interacted proteins ZDHHC5,8,9, resulting in the decrease of the entry efficiencies of SARS-CoV-2 pseudovirus into A549 and Hela cells.

An interesting phenomenon is that the entry efficiencies of SARS-CoV-2 spike-mediated pseudovirus into the two different cell lines A549 and Hela are significantly different. The entry efficiency of SARS-CoV-2 spike-mediated pseudovirus into A549 cells was significantly higher than that into Hela cells, indicating that A549 cells were more susceptible to SARS-CoV-2 pseudovirus infection than Hela cells. It has been reported that the lung is the primary tropism of SARS-CoV-2 [32]. However, whether this phenomenon was linked to the pathogenesis of SARS-CoV-2 in different tissues needs more evidence.

Protein palmitoylation is a dynamic and reversible post-translational modification [10]. The depalmitoylation of ectopically expressed S protein in Hela cells was detected [25], but the enzyme responsible for the depalmitoylation of S protein is unclear. APT1 and APT2 are two main depalmitoylases. It has been reported that APT1 can depalmitoylate the glycoproteins of several enveloped viruses in vitro [33]. APT1 is predominantly localized in mitochondria [34] and APT2 is a cytosolic protein [35]. In this study, the depalmitoylase APT2 that interacted with S protein was detected by co-IP. This strongly implied that APT2 might participate in the depalmitoylation and function of S protein. The mechanism and biological significance of interaction between APT2 and S protein need to more evidence.

## Conclusions

This report confirmed and elucidated the interactions of three host proteins including ZDHHC5, GOLGA7, and APT2 with SARS-CoV-2 S protein, revealing that ZDHHC5 and GOLGA7 played important roles in SARS-CoV-2 pseudovirus entry. The reason why ZDHHC5 or GOLGA7 knockout decreased pseudovirus entry remains unclear. In the current study, the interactions between proteins of pathogen and host provide potential drug targets for antiviral therapy, extending the road to the treatment of infectious diseases, though it has long way to go.

## Data Availability

All data generated or analyzed during this study are included in this published article.

## Abbreviations

ABE: Acyl-biotin exchange
BSA: Bovine serum albumin
BSL2: Biosafety level 2
co-IP: Co-immunoprecipitation
COVID-19: Coronavirus disease 19
EGFP: Enhanced green fluorescent protein
FBS: Fetal bovine serum
HAM: Hydroxylamine
hpi: H post infection
hpt: H post transfection
IFA: Immunofluorescence assay.
IP: Immunoprecipitated protein complexes
NEM: N-ethylmaleimide
NOD1/2: Nucleotide oligomerization domain (NOD)-like receptors 1 and 2
ORF: Open reading frame
PBS: Phosphate-buffered saline
PDZ: PSD-95/Discs-large/ZO-1 homology
PMSF: Phenylmethylsulfonyl fluoride
PVDF: Polyvinylidene difluoride
RFP: Red fluorescent protein
RIPA: Radio immunoprecipitation assay
RLU: Relative light units
SARS-CoV: Severe acute respiratory syndrome coronavirus
SARS-CoV-2: Severe acute respiratory syndrome coronavirus 2
