# Correction to: The interactions of ZDHHC5/GOLGA7 with SARS‑CoV‑2 spike (S) protein and their effects on S protein’s subcellular localization, palmitoylation and pseudovirus entry

**DOI:** 10.1186/s12985-022-01750-0

**Published:** 2022-02-03

**Authors:** Xiao‑Tao Zeng, Xiao‑Xi Yu, Wei Cheng

**Affiliations:** Division of Respiratory and Critical Care Medicine, Respiratory Infection and Intervention Laboratory of Frontiers Science Center for Disease‑Related Molecular Network, State Key Laboratory of Biotherapy, West China Hospital of Sichuan University, Chengdu, 610041 China

## Correction to: Virology Journal (2021) 18:257 https://doi.org/10.1186/s12985-021-01722-w

Following publication of the original article [1], the authors identified an error in the author name of Xiao‑Xi Yu.

The incorrect author name is: Xiao‑Ti Yu.

The correct author name is: Xiao‑Xi Yu.

The author group has been updated above and the original article [1] has been corrected.

## References

[1] Zeng, et al. Virol J. 2021;18:257. 10.1186/s12985-021-01722-w.

